# Safety and use of complementary and alternative medicine in Norway during the first wave of the COVID-19 pandemic using an adapted version of the I-CAM-Q; a cross-sectional survey

**DOI:** 10.1186/s12906-022-03656-4

**Published:** 2022-09-03

**Authors:** Agnete Egilsdatter Kristoffersen, Miek C. Jong, Johanna Hök Nordberg, Esther T. van der Werf, Trine Stub

**Affiliations:** 1 National Research Center in Complementary and Alternative Medicine (NAFKAM), Department of Community Medicine, UiT The Arctic University of Norway, Tromsø, Norway; 2 Regional Cancer Center Stockholm Gotland, Stockholm, Sweden; 3 Divison of Nursing & Dept Physiology & Pharmacology, Karolinska Institutet, Dept Neurobiology, Care Sciences & Society, Stockholm, Sweden; 4 Louis Bolk Institute, Bunnik, the Netherlands; 5 The Consortium for Integrative Medicine and Health (CIZG), Groningen, the Netherlands

**Keywords:** CAM, T&CM, COVID-19, Norway, Complementary therapies, Safety

## Abstract

**Background:**

The COVID-19 pandemic lockdown has had a profound impact on physical and mental well-being throughout the world. Previous studies have revealed that complementary and alternative medicine (CAM) is frequently used for, and can be potential beneficial for strengthening physical mental resilience. The aims of this study were therefore to determine the prevalence and reasons for use of CAM during the first wave of the COVID-19 pandemic among a representative sample of the Norwegian population, and further determine self-reported effects and adverse effects of the CAM modalities used.

**Methods:**

Computer assisted telephone interviews using a COVID-adapted I-CAM-Q questionnaire were conducted with 1008 randomly selected Norwegians aged 16 and above using multistage sampling during April and May 2020 applying age and sex quotas for each area. Frequencies, Pearson’s chi-square tests, Fisher exact tests, and independent sample t-test were used to identify the users of CAM, what they used, why they used it and whether they experienced effect and/or adverse effects of the modalities used, and further to describe differences in sociodemographic factors associated with CAM use. Cronbach's alpha tests were used to test for internal consistency in the different groups of CAM. Significance level was set to *p <* 0.05.

**Results:**

The study revealed that two thirds of the respondents (67%) had used CAM within the first 3 months of the COVID-19 pandemic, in particular CAM modalities that did not involve a provider. Most used were natural remedies and dietary supplement (57%, mainly vitamins and minerals), but self-help practices like yoga and meditation were also widely used (24%). Women used CAM modalities significantly more than men (77% vs. 58%). Most of the respondents found the modalities they used beneficial, and few reported adverse effects of the treatments.

**Conclusions:**

A large proportion of the Norwegian population used CAM during the first wave of the COVID-19 pandemic with high satisfaction and few reported adverse effects. CAM was rarely used to prevent or treat COVID-19, but rather to treat a long-term health condition, and to improve well-being.

## Background

The COVID-19 pandemic lockdown had a profound impact on physical and mental well-being throughout the world [1]. In Norway, a nationwide lockdown was implemented on March 12, 2020 [2]. Kindergarten, schools, and universities were closed and employees who could work from home were instructed to do so. Although the lockdown most likely decreased the spread and harm from COVID-19 before vaccines were available [3], studies describe that these restrictions might have serious mental health effects on the population [4–7], in particular in groups that already were vulnerable [8]. While the COVID-19 pandemic has led to increased activity for parts of health care [9], referral to a wide range of mental health services has decreased [1]. Health care providers working outside established health care services were instructed to close down their clinics and stop offering treatments that included physical contact between provider and client. This led to a lockdown among many complementary and alternative medicine (CAM) providers or reorganisation of their practice to telephone- and video consultations [2] where that could be of help to their clients.

CAM is defined as a group of diverse medical and health care practices and products that are not generally considered part of conventional medicine [4]. Several subcategories of health care-seeking behaviour fall under the definition of CAM: Visits to CAM providers; use of natural remedies and dietary supplements; and different types of self-help practices [5]. CAM is widely used, ranging from 10% [6] to 76% worldwide [7]. CAM is also commonly used in Europe [6], and Scandinavia [8, 10]. In Norway a recently published study demonstrated that 62.2% of the respondents reported to have used CAM within a 12 months period [11]. Most respondents had used natural remedies and dietary supplements (47.7%), followed by self-help practices such as yoga and meditation (29.1%), and consultations with CAM providers (14.7% ) [11].

Studies describing prevalence of CAM use during the COVID-19 pandemic are still sparse, but an Irani study reported 84% use [12], and a Ghanian study reported 85.5% use of CAM during COVID-19 outbreak [13]. In Saudi Arabia 93% of the population reported to use natural or herbal products during the COVID-19 [14] while 56.6% in United Arab Emirates reported use of dietary supplements for prevention or treatment of COVID-19 [15]. A Polish study found increased consumption of dietary supplements during the first wave of the COVID-19 pandemic [16], and in Cameroon did 24.4% of the studied population use herbal medicine for COVID-19 prevention [17].

A previous literature review described that CAM is frequently used to strengthen physical and mental resilience, and describe a possibly direct effect of CAM approaches like vitamin A, D, and mind-body interventions on immune functions and influenza, relevant for people during the COVID-19 pandemic [18]. However, it is not known whether people in Norway are making use of CAM to deal with possible negative health effects of the COVID-19 related restrictions such as weight gain [19], depression, anxieties, economic pressure, and mental illness [20]. Furthermore, people’s access to CAM might have been limited as the CAM providers had to close down their practice during the lockdown the first 3 months of the pandemic.

The aims of this study were therefore to determine the prevalence and reasons for use of CAM during the first wave of the COVID-19 pandemic among a representative sample of the Norwegian population, and further determine self-reported effects and adverse effects of the CAM modalities used.

## Methods

In Norway, a national cross-sectional survey was carried out between April 28 and May 5 2020 using computer-assisted telephone interviews. The survey was conducted in collaboration with the marketing research company Ipsos A/S and had a target sample of 1000 people, representing the Norwegian population of 4.4 million inhabitants aged 16 and above [21].

Ipsos A/S receives general and randomized telephone numbers draw from Bisnode, a private company that receives raw data from hundreds of subcontractors, both public and private. This includes information on names, addresses and income from the Tax Administration, telephone numbers from telecom operators and public data from the Brønnøysund Register Centre that operate many of the country’s most important registers. This feature is checked against Statistics Norway’s figures on population structure by regions, counties and municipalities, as well as gender and age. Number lists are set up accordingly in order to achieve as representative a sample as possible. This requires the formulation of specific quotas based on experience with response rates in different parts of the population.

**Fig. 1:**
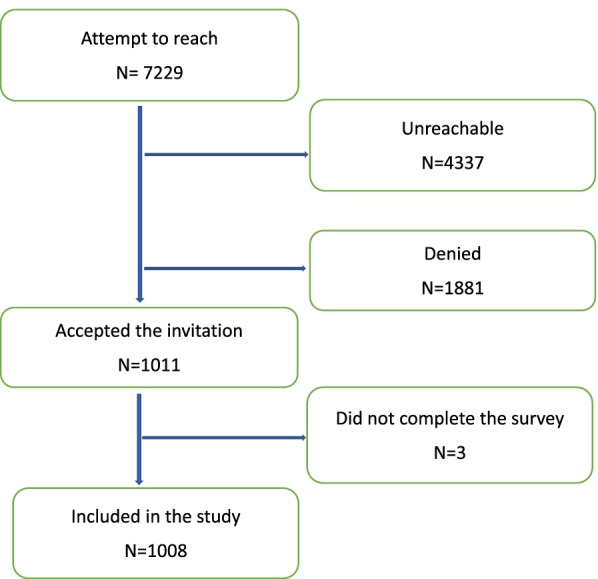
Flowchart of the respondents in the survey

The sample was therefore stratified by sex, age and region of residence and drawn from Norwegian residents aged 16 and above living in private households with a landline telephone or a cell phone using random quota sampling. Up to 7 attempts were made to reach the selected person with the following introduction: *“Good evening, it’s ........ calling from Ipsos. We are conducting a survey, and I would like to ask you some questions. Is that okay?”* for cell phone numbers and *“Good evening, it’s ...….. calling from Ipsos. We are conducting a survey, and I would like to asked some questions to the person who last had a birthday in this household”* for land line numbers. *N =* 4337 were unreachable after 7 calling attempts (Fig. 1). Individuals who were reached and refused participation (*n =* 1881) were considered non-respondents, leading to a response rate of 34.5%. The final sample contained 1008 individuals, 487 women and 521 men (Fig. 1).

### Inclusion and exclusion criteria

Included in the study was Norwegian residents aged 16 and above with a land line or cell-phone number, reachable within seven attempts.

All residents under the age of 16, residents without a land line or cell-phone number, and residents aged 16 and above not reachable after 7 attempts were excluded from the study.

### Survey instrument

A COVID-19 adapted version of the validated survey instrument International Questionnaire to Measure Use of Complementary and Alternative Medicine (I-CAM-Q) [5, 22] was used addressing COVID-19 related use (for prevention or treatment of COVID-19) in addition to the existing options of use; for acute illness, chronic illness, well-being and other, unspecified reasons. The survey consisted of three parts: The first part related to CAM use offered by CAM providers, the second part included questions regarding use of natural remedies and dietary supplements, while the third part included questions related to self-help practices such as mindfulness, yoga, and relaxation techniques. In all parts, the questions referred to use during the last 3 months. For all CAM modalities used, there were follow-up questions regarding benefit of use, and possible adverse effects of the treatment, with the adverse effect question added to the original I-CAM-Q. Demographic characteristics collected were sex, age, level of education, household income and place of residence. All data were anonymously collected and reported.

### Measures

#### Measures of personal characteristics

Age was obtained as an open question and assessed as a continuous variable as well as categorical after being divided into four groups; 16-24 years; 25-39; 40-59 years; and 60 year or more.

Level of education was collected using six categories: 1. Primary school up to 8 years; 2. Primary school up to 10 years; 3. Secondary school; 4. College/university less than 4 years; and 5. College/university 4 years or more. These were merged into primary school (1-2); secondary school (3); and college/university (4-5) in the description of the study population, and further into primary/secondary school (1-3); and college/university (4-5) in the analyses.

Household income was collected using the following categories (NOK < 100,000; 100,000-199,000; 200,000-299,000; 300,000-399,000; 400,000-499,000; 500,000-599,000; 600,000-799,000; 800,000-999,000; 1000,000-1500,000; and more than NOK 1500,000). These categories were merged into low household income (<NOK400,000), middle household income (NOK 400,000-799,000) and high household income (NOK 800,000 or more).

Other personal characteristics included sex (female, male) and residence (merged into the Norwegian regions South-East; South; West; Central (Trøndelag); and North).

#### CAM providers

The measure *consultations with CAM providers* were specified by visits to massage therapists, naprapaths, acupuncturists, coaches, healers, homeopaths, cupping therapists, herbalists, traditional healers, osteopaths, kinesiologists, and other CAM providers. Internal consistency of the CAM providers analysed in this paper was found to be 0.491 using a Cronbach’s alpha test.

The respondents were asked whether they had consulted one or more CAM providers and if yes; reasons, benefit, and possible adverse effects of the treatments.

#### Natural remedies and dietary supplements

Use of natural remedies and dietary supplements were mapped by providing the respondents with a list of remedies organized as *herbs* (ginger, curcumin, garlic, green tea, herbal tea, cranberry, blueberry/blueberry extract, oregano, echinacea, chaga, and other), *vitamins and minerals* (vitamin B, vitamin C, vitamin D, magnesium, calcium, iron, zinc, selenium, and other vitamins and minerals), *homeopathic remedies, Bach flower remedies,* and *dietary supplements* (Omega 3, 6 or 9, protein shake, Q10, and other dietary supplements). The respondents were asked whether they had used one or more of these remedies, and if yes; reasons, benefit, and possible adverse effects of this use. Internal consistency of the natural remedies and dietary supplements analysed in this paper was found to be 0.744 using a Cronbach’s alpha test.

#### Self-help practices

*Use of self-help practices* were measured by specific questions regarding the use of meditation, yoga, qigong, tai chi, relaxation, visualization, mindfulness, lightning process, neuro-linguistic programming (NLP), participation in traditional healing rituals, and other self-help practices. The respondents were asked *if* they had used these self-help practices and further; why, whether they found it to be beneficial, and if they experienced adverse effects. Internal consistency of self-help practices analysed in this paper was found to be 0.579 using a Cronbach’s alpha test.

#### Over-all use of CAM

Over-all-CAM use was measured by calculating the total number of CAM users reported, combining the variables *modalities provided by CAM providers*, *natural remedies and dietary supplements*, and *self-help practices.* Internal consistency of over-all use of CAM was found to be 0.685 using a Cronbach’s alpha test.

#### Reasons, benefit, and possible adverse effects of the treatments

The reason(s), benefits and adverse effects of the treatments were measured through the following questions following each modality used:

*Please indicate the reason why you used the therapy* with the response options: 1. *For an acute illness/condition that lasted less than a month*; 2. *To treat a long-term health condition (one that lasted more than a month) or its symptoms*; 3. *To improve well-being*; 4. *For prevention of COVID-19*; 5. *To treat COVID-19-related symptoms*; and 6. *Other reasons*.

Possible benefits of the modality were measured with the question: *How helpful was it to you*? with the response options: 1. *Very*; 2. *Somewhat*; 3. *Not at all*; and 4. *Don’t know* following each modality.

The following question was added to the original I-CAM-Q for each CAM modality: *Did you experience any adverse effects of the treatment*? with the response options: 1. *Yes*; 2. *No*; and 3. *Don’t know*.

### Statistics

With a margin of error of 5%, a confidence level of 95%, and a heterogeneity of 50%, we needed a minimum sample of *n =* 384 to represent the adult Norwegian population (16 years and above) of 4.4 million [21] for adequate study power [19]. As increased sample size is associated with decreased sampling error and is more likely to represent the population [20], the sample size was set to *n =* 1000. Descriptive statistics were carried out using Statistical Package for Social Sciences (SPSS) v. 28.0. Descriptive statistics are the basic measures used to describe survey data [23], and have an important role in medical research [24]. Descriptive studies often represent the first scientific approach in new areas of inquiry. Descriptive statistics only describe the existing distribution of variables, without regard to causal or other hypotheses [25]. Good descriptive reporting answers the five basic W questions: who, what, why, when, and where [24]. As when (last 3 months), and where (Norway) already were defined by the researchers, frequencies, Pearson’s chi-square tests, Fisher exact tests, and independent sample t-tests were used to identify the users of CAM (who), what they used, why they used it and whether they experienced effect and/or adverse effects of the modalities, and further to describe differences in sociodemographic factors (age, education level, household income) between CAM users and non-CAM users with a significance level set to *p <* 0.05. The specific test used for each analysis will be described in the tables. A Cronbach’s alpha test of internal consistency were run for the different groups of CAM analyzed in this paper (CAM providers, natural remedies and dietary supplements, and self-help practices).

## Results

### Basic characteristics of the respondents

The survey consisted of slightly more men than women (51.7% vs. 48.3%), with a mean age of 44.9 and 48.0 years respectively (*p =* 0.009). The majority of respondents had high income (41.7%), a college or university education (53.5%) and were living in the South-Eastern part of Norway (50.7%). The men had higher income (*p =* 0.017) and lower education (*p =* 0.002) compared to women (Table 1).

**Table 1:** Basic characteristics of the respondents

	Total		Women		Men		
	%	(*n =* 1008)	%	(*n =* 487)	%	(*n =* 521)	*p*-value
**Gender**							
Men	51.7	(521)					
Women	48.3	(487)					
**Age**							
Mean age (years) (SD)	46.4 (SD18.678) (1008)		48.0 (SD18.959) (487)		44.9 (SD18.305) (521)		0.009^b^
18-24 years	15.9	(160)	14.8	(72)	16.9	(88)	0.059^a^
25-39 years	25.1	(253)	22.0	(107)	28.0	(146)	
40-59	32.7	(330)	34.4	(168)	31.1	(162)	
≥ 60 years	26.3	(265)	28.7	(140)	24.0	(125)	
**Household income**^**c**^							0.017^a^
Low	10.5	(106)	11.5	(56)	9.6	(50)	
Middle	26.8	(270)	25.5	(124)	28.0	(146)	
High	41.7	(420)	38.4	(187)	44.7	(233)	
Did not want to answer	6.2	(62)	6.2	(30)	6.1	(32)	
Did not know	14.9	(150)	18.5	(90)	11.5	(60)	
**Years of Education**							0.002^a^
Primary school	11.0	(111)	11.1	(54)	10.9	(57)	
Secondary school	35.5	(358)	30.2	(147)	40.5	(211)	
College/university	53.5	(539)	58.7	(286)	48.6	(253)	
**Region of residence**							0.929^a^
South-East	50.7	(511)	51.1	(249)	50.3	(262)	
South	5.7	(57)	5.3	(26)	6.0	(31)	
West	25.3	(255)	26.1	(127)	24.6	(128)	
Central (Trøndelag)	8.7	(88)	8.4	(41)	9.0	(47)	
North	9.6	(97)	9.0	(44)	10.2	(53)	

^a^Pearson chi-square test; ^b^Independent samples t-test; ^c^Low (<NOK400,000/EUR 40,000), middle (NOK 400,000-799,000/EUR40,000-79,900), and high (≥ NOK 800,000/EUR 80,000)

### CAM use

A total of 67.2% of the respondents had used CAM during the first 3 months of the COVID-19 pandemic, more women (77.4%) than men (57.6%, *p <* 0.001, Table 2). Less than 8% of the survey respondents had consulted CAM providers (Table 2). Most frequently used were natural remedies and dietary supplements (57%), followed by self-help practises (24.2%).

**Table 2:** Use of CAM among female and male respondents during the first 3 months of the COVID-19 pandemic

	Total	Women	Men	*p*-value
	% (n)	% (n)	% (n)	
**Consultations with CAM providers**	**7.8 (79)**	**10.1 (49)**	**5.5 (30)**	**0.011** ^a^
**Use of natural remedies and dietary supplements**	**57.0 (575)**	**66.7 (325)**	**48.0 (250)**	**<0.001** ^a^
Herbs	14.9 (150)	19.1 (93)	10.9 (57)	<0.001^a^
Vitamins and minerals^c^	43.1 (434)	52.4 (255)	34.4 (179)	<0.001^a^
Homeopathic remedies	1.3 (13)	1.0 (5)	1.5 (8)	0.474^a^
Bach flower remedies	0.4 (4)	0.8 (4)	0.0 (0)	0.054^b^
Other dietary supplements	23.7 (239)	26.5 (129)	21.1 (110)	0.045^a^
**Use of self-help practices (yoga, meditation etc.)**	**24.2 (244)**	**32.4 (158)**	**16.5 (86)**	**<0.001** ^a^
**Total use of CAM (providers, supplements and/or self-help)**	**67.2 (677)**	**77.4 (377)**	**57.6 (300)**	**<0.001** ^a^

^a^Pearson Chi-Square test

^b^Fisher exact test

^c^Vitamins and minerals other than multi-vitamin tablets/mixture

### Consultation with CAM providers

CAM providers were consulted by 7.8% of the respondents, 10.1% of the women and 5.5% of the men (*p =* 0.011) during the first 3 months of the COVID-19 pandemic (Table 2). Massage therapists (3%), naprapaths (1.5%), psychotherapists (1.4%), and acupuncturists (1.2%) were the most visited CAM providers. Most of the respondents found the treatment helpful, ranging from 91.7% (acupuncturists) to 100% (naprapaths, psychotherapists, and reflexologists) mainly for chronic complaints. One had consulted a traditional healer for preventing COVID-19. Men and women consulted CAM providers to a similar degree except for massage therapists who were more frequently visited by women (4.1%) compared to men (1.9%, *p =* 0.041, Table 3).

**Table 3:** The most commonly used CAM modalities during the first 3 months of the COVID-19 pandemic, reason(s) for use, and self-perceived benefit(s)

					Reason(s) for use^c^					
	Total	Women	Men	*p*-value	Acute illness	Long-term illness	Improvement of wellbeing	Prevent/treat COVID-19	Other	Very or somewhat helpful
	% (n)	% (n)	% (n)		% (n)	% (n)	% (n)	% (n)	% (n)	% (n)
**Consultations with CAM providers**										
Massage therapists	3.0 (30)	4.1 (20)	1.9 (10)	**0.041** ^a^	0.0 (0)	40.0 (12)	36.7 (11)	0.0 (0)	23.3 (7)	93.3 (28)
Naprapaths	1.5 (15)	1.6 (8)	1.3 (7)	0.695^a^	13.3 (2)	73.3 (11)	0.0 (0)	0.0 (0)	13.3 (2)	100 (15)
Psychotherapists	1.4 (14)	1.4 (7)	1.3 (7)	0.899^a^	7.1 (1)	64.3 (9)	21.4 (3)	0.0 (0)	7.1 (1)	100 (14)
Acupuncturists	1.2 (12)	1.2 (6)	1.2 (6)	0.906^a^	16.7 (2)	50.0 (6)	8.3 (1)	0.0 (0)	25.0 (3)	91.7 (11)
Reflexologists	1.0 (10)	0.8 (4)	1.2 (6)	0.754^b^	10.0 (1)	50.0 (5)	30.0 (3)	0.0 (0)	10.0 (1)	100 (10)
**Use of natural remedies and dietary supplements**										
Vitamin D	22.6 (228)	29.4 (143)	16.3 (85)	**<0.001** ^a^	3.5 (8)	21.5 (49)	32.9 (75)	1.8 (4)	45.2 (103)	72.4 (165)
Omega 3, 6 or 9	18.8 (190)	21.8 (106)	16.1 (84)	**0.022** ^a^	0.5 (1)	10.0 (19)	48.4 (92)	2.1 (4)	44.7 (85)	62.6 (119)
Vitamin C	16.5 (166)	18.7 (91)	14.4 (75)	0.066^a^	12.0 (20)	5.4 (9)	38.6 (64)	5.4 (9)	44.6 (74)	64.4 (107)
Magnesium	10.8 (109)	14.6 (71)	7.3 (38)	**<0.001** ^a^	0.0 (0)	31.2 (34)	23.9 (26)	2.8 (3)	41.3 (45)	75.2 (82)
Iron	5.5 (55)	8.8 (43)	2.3 (12)	**<0.001** ^a^	5.5 (3)	23.6 (13)	32.7 (18)	1.8 (1)	43.6 (24)	90.9 (50)
Calcium	5.5 (55)	8.2 (40)	2.9 (15)	**<0.001** ^a^	0.0 (0)	45.5 (25)	27.3 (15)	3.6 (2)	27.3 (15)	67.3 (37)
Ginger	5.0 (50)	5.7 (28)	4.2 (22)	0.265^a^	12.0 (6)	14.0 (7)	34.0 (17)	4.0 (2)	48.0 (24)	74.0 (37)
Herbal tea	4.2 (42)	6.0 (29)	2.5 (13)	**0.006** ^a^	7.1 (3)	11.9 (5)	52.4 (22)	2.4 (1)	28.6 (12)	64.3 (27)
Garlic	4.0 (40)	4.3 (21)	3.5 (19)	0.589^a^	12.5 (5)	7.5 (3)	27.5 (11)	5.0 (2)	60.0 (24)	67.5 (27)
Green tea	3.9 (39)	4.7 (23)	3.1 (16)	0.174^a^	0.0 (0)	5.1 (2)	66.7 (26)	2.6 (1)	33.3 (13)	56.4 (24)
**Self-help practices**										
Yoga	13.2 (133)	20.5 (100)	6.3 (33)	**<0.001** ^a^	2.3 (3)	13.5 (18)	54.9 (73)	0.0 (0)	36.8 (49)	93.3 (83)
Meditation / mindfulness	10.9 (110)	14.0 (68)	8.1 (42)	**0.003** ^a^	2.7 (3)	15.5 (17)	67.3 (74)	1.8 (2)	29.1 (32)	93.8 (103)
Relaxation	7.8 (79)	11.9 (58)	4.0 (21)	**<0.001** ^a^	6.3 (5)	29.1 (23)	55.7 (44)	0.0 (0)	19.0 (15)	98.7 (78)
Visualization	3.3 (33)	4.3 (21)	2.3 (12)	0.073^a^	6.1 (2)	12.1 (4)	54.5 (18)	0.0 (0)	39.4 (13)	97.0 (32)

^a^Pearson Chi-Square test

^b^Fisher exact test

^c^Multiple choices possible so the total sum might exceed 100%

The respondents who had consulted CAM providers were mainly middle aged, with primary/secondary education, high income, and living in the South-Eastern part of Norway. They did; however not differ from respondents *not* consulting CAM providers in regard to these factors (Table 4, *p* > 0.217). Similar characteristics were found for both women and men apart from educational level were the women who consulted CAM providers were more likely to have college or university education (61.2%) compared to men (23.3%, *p =* 0.001, Table 5).

**Table 4:** Characteristics of the users and non-users of CAM during the first 3 months of the COVID-19 pandemic

	CAM providers			Natural remedies and dietary supplements			Self-help practices			Total CAM use		
	Yes	No		Yes	No		Yes	No		Yes	No	
	% (n)	% (n)	*p*-value	% (n)	% (n)	*p*-value	% (n)	% (n)	*p*-value	% (n)	% (n)	*p*-value
**Sex**			0.011^a^			<0.001^a^			<0.001^a^			<0.001^a^
Female	62.0 (49)	47.1 (348)		56.5 (325)	37.4 (162)		64.8 (158)	43.1 (329)		55.7 (377)	33.2 (110)	
Male	38.0 (30)	52.9 (491)		43.5 (250)	62.6 (271)		35.2 (86)	56.9 (435)		44.3 (300)	66.8 (221)	
**Age**			0.302^a^			0.068^a^			<0.001^a^			0.324^a^
16-24 years	8.9 (7)	16.5 (153)		15.8 (91)	15.9 (69)		20.9 (51)	14.3 (109)		16.5 (112)	14.5 (48)	
25-39 yeras	29.2 (23)	24.8 (230)		23.5 (135)	27.3 (118)		32.8 (80)	22.6 (173)		25.4 (172)	24.5 (81)	
40-59 years	36.7 (29)	32.4 (301)		31.3 (180)	34.6 (150)		34.0 (83)	32.3 (247)		30.9 (209)	36.6 (121)	
60 years or more	25.3 (20)	26.4 (245)		29.4 (169)	22.2 (96)		12.3 (30)	30.8 (235)		27.2 (184)	24.5 (81)	
Mean (SD)	47.9 (16.41)	46.3(18.86)	0.463^c^	47.9 (16.41)	45.0(18.04)	0.035^c^	40.0(16.19)	48.5(18.96)	<0.001^c^	46.4(18.89)	46.6(18.26)	0.869^c^
**Education**			0.218^a^			0.652^a^			0.001^a^			0.179^a^
Primary/secondary	53.2 (42)	46.0 (427)		45.9 (264)	47.3 (205)		37.3 (91)	49.5 (378)		45.1 (305)	49.5 (164)	
College/university	46.8 (37)	54.0 (502)		54.1 (311)	52.7 (228)		62.7 (153)	50.5 (386)		54.9 (372)	50.5 (167)	
**Household income**			0.726^a^			0.132^a^			0.489^a^			0.249^a^
Low	15.9 (10)	13.1 (96)		15.3 (70)	10.7 (36)		15.7 (31)	12.5 (75)		14.7 (79)	10.4 (27)	
Middel	30.2 (19)	34.2 (251)		34.1 (156)	33.7 (114)		31.8 (63)	34.6 (207)		33.3 (179)	35.1 (91)	
High	54.0 (34)	52.7 (386)		50.7 (232)	55.6 (188)		52.5 (104)	52.8 (316)		52.0 (279)	54.4 (141)	
**Region of residence**			0.556^b^			0.563^a^			0.157^a^			0.518^a^
East	58.2 (46)	50.1 (465)		51.5 (296)	49.7 (215)		54.5 (133)	49.5 (378)		52.1 (353)	47.7 (158)	
South	2.5 (2)	5.9 (55)		5.2 (30)	6.2 (27)		4.5 (11)	6.0 (46)		5.0 (34)	6.9 (23)	
West	20.3 (16)	25.7 (239)		26.4 (152)	23.8 (103)		20.1 (49)	27.0 (206)		25.3 (171)	25.4 (84)	
Central (Trøndelag)	8.9 (7)	8.7 (81)		8.2 (47)	9.5 (41)		10.7 (26)	8.1 (62)		8.1 (55)	10.0 (33)	
North	10.1 (8)	9.6 (89)		8.7 (50)	10.9 (47)		10.2 (25)	9.4 (72)		9.5 (64)	10.0 (33)	

^a^Pearson chi-square test

^b^Fisher exact test

^c^Independent sample t-test

**Table 5:** Differences between women and men using CAM during the first 3 months of the COVID-19 pandemic

	CAM providers			Natural remedies and dietary supplements			Self-help practices			Total CAM use		
	Women	Men		Women	Men		Women	Men		Women	Men	
	% (n)	% (n)	*p*-value	% (n)	% (n)	*p*-value	% (n)	% (n)	*p*-value	% (n)	% (n)	*p*-value
**Age**			0.650^b^			0.152^a^			0.010^a^			0.103^a^
16-24 years	8.2 (4)	10.0 (3)		13.8 (45)	18.4 (46)		19.0 (30)	24.4 (21)		15.1 (57)	18.3 (55)	
25-39 yeras	24.5 (12)	36.7 (11)		21.5 (70)	26.0 (65)		27.8 (44)	41.9 (36)		22.8 (86)	28.7 (86)	
40-59 years	40.8 (20)	30.0 (9)		34.2 (111)	27.6 (69)		36.7 (58)	29.1 (25)		34.0 (128)	27.0 (81)	
60 years or more	26.5 (13)	23.3 (7)		30.5 (99)	28.0 (70)		16.5 (26)	4.7 (4)		28.1 (106)	26.0 (78)	
Mean (SD)	50.1 (16.40)	44.4 (16.09)	0.136^c^	48.8 (19.04)	45.8 (19.06)	0.063^c^	42.4 (16.74)	35.6 (14.17)	0.002^c^	47.6 (18.91)	44.8 (18.78)	0.055^c^
**Education**			0.001^a^			0.001^a^			0.006^b^			<0.001^a^
Primary/secondary	38.8 (19)	76.7 (23)		39.7 (129)	54.0 (135)		31.0 (49)	48.8 (42)		38.5 (145)	53.3 (160)	
College/university	61.2 (30)	23.3 (27)		60.3 (196)	46.0 (115)		69.0 (109)	51.2 (44)		61.5 (232)	46.7 (140)	
**Household income**			0.342^b^			0.703^a^			0.416^a^			0.587^a^
Low	20.0 (8)	8.7 (2)		16.5 (41)	13.9 )29)		18.0 (24)	10.8 (7)		16.2 (47)	13.0 (32)	
Middel	25.0 (10)	39.1 (9)		32.9 (82)	35.4 (74)		30.8 (41)	33.8 (22)		33.0 (96)	33.7 (83)	
High	55.0 (22)	52.2 (12)		50.6 (126)	50.7 (106)		51.1 (68)	55.4 (36)		50.9 (148)	53.3 (131)	
**Region of residence**			0.213^b^			0.614^a^			0.365^b^			0.477^a^
East	63.3 (31)	50.0 (15)		52.0 (169)	50.8 (127)		57.0 (90)	50.0 (43)		53.3 (201)	50.7 (152)	
South	2.0 (1)	3.3 (1)		4.0 (13)	6.8 (17)		2.5 (4)	8.1 (7)		3.7 (14)	6.7 (20)	
West	20.4 (10)	20.0 (6)		27.4 (89)	25.2 (63)		19.6 (31)	20.9 (18)		26.0 (98)	24.3 (73)	
Central (Trøndelag)	10.2 (5)	6.7 (2)		7.7 (25)	8.8 (22)		10.8 (17)	10.5 (9)		8.0 (30)	8.3 (25)	
North	4.1 (2)	20.0 (6)		8.9 (29)	8.4 (21)		10.1 (15)	10.5 (9)		9.0 (34)	10.0 (30)	

^a^Pearson chi-square test

^b^Fisher exact test

^c^Independent sample t-test

### Natural remedies and dietary supplements

More than half of the respondents (57%) had used natural remedies and dietary supplements during the first 3 months of the COVID-19 pandemic (Table 2), mostly reported to be helpful, ranging from 56.4% (green tea) to 90.9% (iron, Table 3). Natural remedies and dietary supplements were mainly used for well-being or other reasons, only 1.5% (*n =* 15) used natural remedies and dietary supplements to treat or prevent COVID-19; vitamin C (*n =* 9), vitamin D (*n =* 4), Omega 3, 6 or 9 fatty acid (*n =* 4), and magnesium (*n =* 3). Regardless of reasons for use were vitamin D (22.6%), Omega 3, 6 or 9 (18.8%), and vitamin C (16.5%) most commonly reported by both men and women (Table 3). Women used significantly more natural remedies and dietary supplements compared to men (66.7% vs. 48.0%, *p <* 0.001, Table 2). Besides from being female, the users of natural remedies and dietary supplements were older than the non-users (47.5 years vs. 45.0 years, *p =* 0.035, Table 4). Education and household income were similar among users and non-users of natural remedies and dietary supplements (Table 4, *p* > 0.132), but women were more likely to have college or university education compared to the male users of natural remedies and dietary supplements (60.3% vs. 46.0%, *p =* 0.001) and were somewhat older (48.8 vs. 45.8 years); however not at a significant level (*p =* 0.063, Table 5).

### Self-help practices

Self-help practices were used by 24.2% (*n =* 244) of the respondents during the first 3 months of the COVID-19 pandemic (Table 2), the majority found the self-help practises helpful ranging from 93.3% (yoga) to 98.7% (relaxation, Table 3). The self-help practices were mainly used to improve well-being, only 3 (2 meditation/mindfulness and 1 qigong) had used this to prevent or treat COVID-19 (Table 3). Twice as many women (32.4%) than men (16.5%) used self-help practices (*p <* 0.001, Table 2). The most frequently used self-help practice among women was yoga (20.5%), while meditation/mindfulness (8.1%) was the most used self-help practice among men (Table 3). The respondents involved in self-help practices were younger, and more likely to have a college/university education compared to those who did not partake in such practices (Table 4). Women reporting self-help practices were more likely to have university education compared to men (69.0% vs. 51.2%, *p =* 0.006) and were somewhat older (42.4 vs. 35.6 years, *p =* 0.002, Table 5).

### Safety of CAM

Most of the respondents (95%) used CAM without experiencing adverse effects of the treatment. Five percent (*n =* 34); however reported a total of 42 adverse effects. Four of the respondents (*n =* 4) experienced adverse effects after consultations with CAM providers; 2 from acupuncture, 1 from traditional healing and 1 from cupping.

Twenty-one (*n =* 21) respondents reported 27 adverse effects from natural remedies and dietary supplements; 4 from garlic, 4 from iron, 4 from Omega 3,6 or 9, 2 from protein shake, 2 from magnesium, 2 from ginger, 2 from high doses of vitamin C, 2 from zinc, 1 from vitamin D, 1 from vitamin B, 1 from herbal tea, 1 from lemon, and 1 from calcium.

Finally, eleven (*n =* 11) respondents report 11 adverse effects from self-help practices; 9 from yoga, 1 from meditation, and 1 from relaxation. In 32 of the 42 cases (76.2%), adverse effects were reported even though the respondents found the modalities to be very or somewhat helpful.

## Discussion

A total of 67.2% of the participants had used CAM within the first 3 months of the COVID-19 pandemic. Most used were natural remedies and dietary supplement (57.0%) followed by self-help practices (24.2%) and modalities received by CAM providers (7.8%). Women used CAM more often than men (77.4% vs. 57.6%), and while users of self-help practices tended to be younger and more likely to have college and university education, were users of natural remedies and dietary supplement older than the non-users. Most of the respondents found the modalities they used helpful, and few reported adverse effects of the treatments.

The over-all CAM use of 67.2% in the current study is somewhat lower than what was found in Iran where 84% of the survey participants reported to have used at least one type of CAM during the COVID-19 outbreak [12]. The higher use in Iran might be due to cultural differences and the fact that they included prayer in their definition of CAM, used by more than half of the participants. In accordance with the current study, did they find natural remedies and dietary supplements to be the most commonly used CAM therapies during the COVID-19 outbreak [12]. Our findings are also lower than what was found in Ghana where 85.5% of the participants reported to have used CAM during the COVID-19 pandemic period [13]. One of the reasons for the higher use in Ghana might be the fact that the Ghanaian study was conducted a year later, measuring CAM use in a longer period of time and at a stage where the pandemic was more widespread in regard to number of people infected. Traditional medicine also has a strong position in Ghana due to high availability, easy access, low costs, and perception towards illness [13].

The findings of 67.2% CAM use during the first 3 months of the COVID-19 pandemic is 5% higher than the overall CAM use (62.2%) reported in a similar population 1 year earlier [11]. In particular, it appeared that the use of natural remedies and dietary supplements had increased during the COVID-19 pandemic compared to the year before (57.0% versus 47.4%, respectively) [11]. The reason for this, might however be due to the inclusion of Omega 3, 6 and 9 fatty acids in the current study and not in the previous study as Omega 3,6 and 9 fatty acids were among the most commonly used natural remedies in the current study. Increased use of natural remedies and dietary supplements are; however in line with a recently published study from Poland demonstrating increased consumption of dietary supplements like vitamins C and D, zinc, and omega-3 during the first wave of the COVID-19 pandemic [16]. In this Polish study, 13% of respondents started supplementation during the pandemic because they wanted to improve the immunity and/or to be protected against COVID-19 [16]. High use of natural remedies and dietary supplements were also found in Saudi Arabia where 93% of the population used natural or herbal products to improve their immunity during the COVID-19 pandemic with an increase in people using natural remedies or herbal products regularly from 7.3% before the pandemic to near 46% during the pandemic [14]. High use of dietary supplements was also found in United Arab Emirates where 56.6% of the participants used dietary supplements for prevention or treatment of COVID-19 [15]. In contrast to these findings, we found that only a small proportion (1.5%) of people living in Norway used natural remedies, vitamins, minerals and dietary supplements to prevent or treat COVID-19. Therefore, the observed 10% increase in supplementation in the current study compared to a year earlier cannot be explained by the fact that more people in Norway were using supplements to specifically prevent or treat COVID-19. Rather, this increased usage seems to be an expression of a wish to actively contribute to increased general well-being, to boost immunity and/or activate health-promoting pathways [18] despite the fact that one of the most frequently used dietary supplements, Omega 3 fatty acid was associated with a lower risk of COVID-19 infections in the United Kingdom, United States, and Sweden [26].

While supplementation increased, it became apparent that self-help practices (24.2% versus 29.1%) and CAM received from providers (7.8% versus 14.2%) had decreased compared to the year before [11]. This decrease in self-help practices and consultations with CAM provider is most likely caused by restricted access to fitness/yoga centers during the nationwide lockdown, and to the fact that CAM providers had to close down their clinics [2]. Altogether, it seems that the overall need of CAM treatment among Norwegian citizens has not profoundly changed by the COVID-19 pandemic. However, a shift has occurred in the type of CAM modalities used which may signify a willingness to actively participate and contribute to one’s health and wellness. If one CAM modality is not accessible, people may just choose another one.

Even though consultations with CAM providers decreased by almost 50% during the first wave of the COVID-19 lockdown [11], 7.8% of the respondents still appeared to have had consulted CAM providers during the lockdown, with good perceived effect and low risk. This finding indicates that some CAM providers managed to reorganize their practice to non-physical consultations (online/telephone consultations) [2]. Likewise, regarding the use of self-help practices, although decreased, almost a quarter of Norwegian citizens continued to use these techniques during the lockdown. Previous research has shown that practicing yoga may lower depression, anxiety, and stress, and increase general wellbeing and peace of mind during the COVID-19 lockdown [27, 28]. Furthermore, it has been reported that mindfulness may increase well-being and provide a tool for people to deal with stressful situations such as the COVID-19 pandemic [28, 29]. A systematic review has in addition revealed that acupuncture, traditional Chinese medicine (TCM), relaxation, and qigong were found to improved psychological symptoms like depression, anxiety, stress, sleep quality, negative emotions, and quality of life in patients already infected with COVID-19, and further improved physical symptoms like inflammation, chest pain, and respiratory function [30].

A total of 5% of the respondents reported adverse effects of the treatment(s). As the respondents who reported adverse effects (*n =* 42) mostly found CAM beneficial (*n =* 32), the adverse effects found in this study might have been minor and transient [31]. In addition, many patients who use CAM believe that healing crisis (you get worse before getting better) is a part of the healing process [32]. This may also be part of the explanation why reported adverse effects from natural remedies are generally low [33]. According to Deng et al. [34], patients who use CAM must make a judgement of risk versus benefit of a modality. If a CAM modality have solid evidence of effect together with low risk profile, the more likely it is that patients will use the modality. On the other hand, if the risk profile (adverse effects) is greater than the beneficial effect, then it is less likely that patients will use it. The patient must weigh the beneficial effect against risk of the modality continuously, and that is what the respondents in our study seems to have done.

The current study reported that women used CAM significantly more than men. These findings are in line with previous studies on CAM use in Norway [11, 35] and other studies in European countries unequivocally demonstrating that CAM use is more common in women [36–40].

### Strengths and limitations of the study

The main findings should be interpreted in light of the strengths and limitations of this study. A strength was the fact that the survey was conducted in a week’s time and within a crucial, lockdown period during the first wave of the COVID-19 pandemic in Norway. Another strength was that a similar study was conducted in the same population 1 year before the outbreak of the pandemic [11]. However, the observed differences between CAM use during the first wave of the pandemic and the year before should be interpreted with the knowledge that the studies used different time frame of CAM use, and slightly different list of therapies. In the current study CAM use within the last 3 months was reported, whereas this was 12 months in the study conducted 1 year previously [11]. This might have influenced the lower number of respondents reporting consultations with CAM providers and use of self-help practices in the current study. Another limitation of the study is that the type of adverse effects is not known. We do not know if they were mild, moderate or severe, or if it was direct or indirect adverse effects [41]. The main limitation of this study was; however, the rather low response rate of 34.5% that might influence the generalizability of the findings. On the other hand, the fact that the sample used was stratified by sex, age and region of residence increases the representativeness of the target population. The validity of self-reported data with a recall of 3 months can also be questioned, though the agreement between self-reported data and registered data is generally high [42].

### Implication of the findings

Despite few reports of adverse effects from natural remedies and dietary supplements, the increased use of these modalities under the COVID-19 pandemic poses further challenges regarding safe use. Encouraged by social media and online marketing, people often buy natural remedies and dietary supplements online and use them without interference of a healthcare professional [43]. Main safety concerns regarding self-use of natural remedies and dietary supplements are their quality, possible interactions with other drug-related treatments [44, 45], and contamination with toxic compounds [46]. National strategies to identify, monitor, and reduce the occurrence of adverse events associated with self-use of CAM are therefore warranted. These strategies may include the development of an online registry system for the reporting of adverse events associated with CAM use in Norway [47], and to educate and inform consumers, patients and healthcare professionals about the potential risks associated with natural remedies and dietary supplements.

It remains to be investigated whether the observed shift toward increased use of natural remedies and dietary supplements during the first wave is maintained under the whole period of the COVID-19 pandemic. The extent to which these CAM modalities may support in the prevention of COVID-19 also remains to be investigated. A first observational study has demonstrated that supplementation with probiotics, omega-3 fatty acid, multivitamin or vitamin D supplements is modest but significantly associated with a lower risk of testing positive for SARS-CoV-2 in women, but not in men [48], and a systematic review revealed that TCM, relaxation, and qigong improved physical symptoms like inflammation, chest pain, and respiratory function in addition to psychological symptoms, and quality of life in patients already infected with COVID-19 [30]. However, controlled high quality studies are needed in order to confirm these results.

## Conclusion

A large proportion of the Norwegian population used CAM during the first wave of the COVID-19 pandemic with high satisfaction and few reported adverse effects. CAM was rarely used to prevent or treat COVID-19, but rather to treat a long-term health condition, and to improve well-being.

## Data Availability

The dataset this paper has been based on has not been deposited in any repository. All dataset and materials are available from the corresponding author upon reasonable request.

## Abbreviations

COVID-19: Coronavirus Disease 2019
CAM: Complementary and Alternative Medicine
I-CAM-Q: International Questionnaire to measure use of Complementary and Alternative Medicine
TCM: Traditional Chinese Medicine
